# Activation of X-Succinate Synthases for Fumarate Hydroalkylation Using an In Vitro Activation Method

**DOI:** 10.21769/BioProtoc.5357

**Published:** 2025-06-20

**Authors:** Anshika Vats, Shukurah Anas, Ankush Chakraborty, Jian Liu, Jimyung Ryu, Sara M. Mann, Mary C. Andorfer

**Affiliations:** Department of Chemistry, Michigan State University, East Lansing, MI, USA

**Keywords:** Glycyl radical enzyme (GRE), Benzylsuccinate synthase (BSS), 4-isopropylbenzylsuccinate synthase (IBSS), Fumarate addition, Anaerobic hydrocarbon degradation, Radical SAM activating enzyme

## Abstract

X-succinate synthase enzymes (XSSs) are a class of glycyl radical enzymes (GREs) that play a pivotal role in microbial anaerobic hydrocarbon degradation. They catalyze the addition of hydrocarbons to fumarate using a protein-based glycyl radical, which must first be installed by a radical *S*-adenosylmethionine (rSAM) activating enzyme (AE). Once activated, XSS enzymes can undergo multiple catalytic cycles, forming C(sp^3^)–C(sp^3^) bonds with high stereoselectivity—a feature that highlights their potential as asymmetric biocatalysts. Due to the insolubility of XSS-AEs when heterologously expressed in *Escherichia coli*, studies have relied on in vivo radical installation protocols. Although these methods have illuminated fundamental details of XSS mechanisms, the inability to install a glycyl radical in vitro has limited biochemical studies and biotechnological advances using these enzymes. Here, we describe an in vitro protocol for reconstituting the activity of benzylsuccinate synthase (BSS), an XSS that catalyzes the addition of toluene to fumarate to form *R*-benzylsuccinate. To enable in vitro glycyl radical installation, we identified a soluble homolog via genome mining: 4-isopropylbenzylsuccinate synthase activating enzyme (IbsAE). IbsAE was expressed in *E. coli* and anaerobically purified in moderate yields (6–8 mg of protein per liter of culture); herein, we outline the expression and anaerobic purification of both IbsAE and BSS proteins. We describe a reproducible method for in vitro glycyl radical installation using these recombinant proteins and provide guidance on quantifying radical formation. Our optimized protocol consistently achieves 30%–50% radical installation, comparable to other in vitro GRE activations. Lastly, we demonstrate the application of this protocol for in vitro hydroalkylation reactions, achieving high assay yields (89%–97%). This protocol enables biochemical experiments that were previously challenging using cell extracts and accelerated advancements in XSS engineering and use in biocatalysis.

Key features

• Builds upon the method described by Andorfer et al. [1] to thoroughly describe in vitro activation and hydroalkylation using glycyl radical enzymes.

• Useful for studying substrate scope and determining the stereoselectivity of XSS-catalyzed reactions with non-native substrates.

• Can serve as a template for the reconstitution of activity for other XSS enzymes.

• Describes protein production through hydroalkylation steps, which take approximately 6–7 days to complete, given that expressions and purifications are performed in parallel.

## Graphical overview


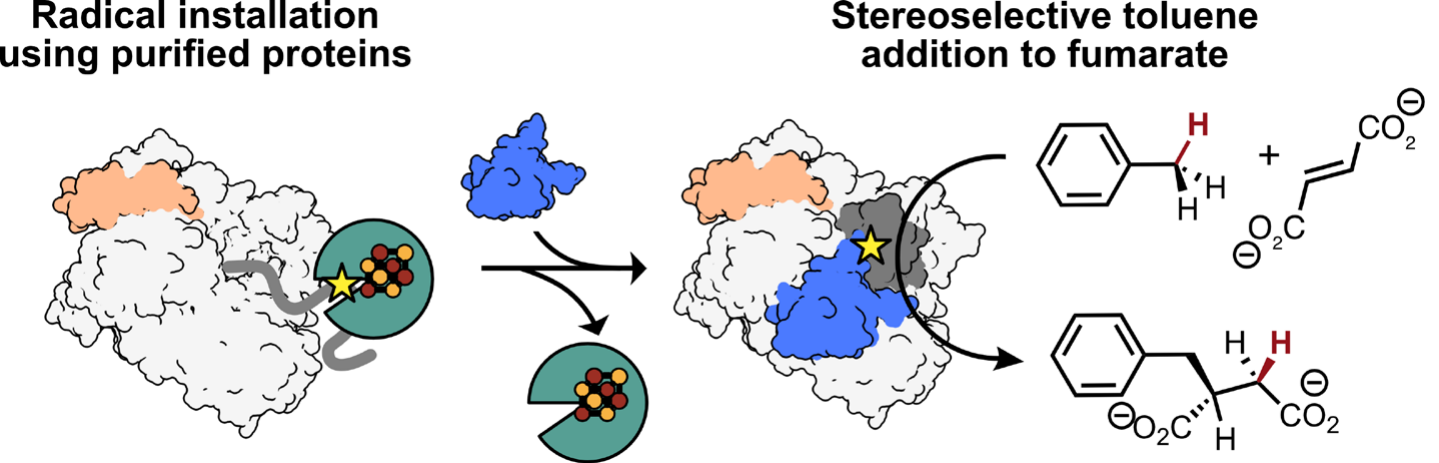


## Background

Hydrocarbons, aside from serving as the major feedstock of the chemical industry, are also the primary constituents of natural gas and crude oil. The efficient and selective activation of C–H bonds within these hydrocarbons can be used as a tool for bioremediation as well as a method to convert low-cost hydrocarbon feedstocks into value-added products [2]. X-succinate synthases (XSSs), a class of glycyl radical enzymes (GREs), can catalyze this initial C–H activation of hydrocarbons to stereoselectively hydroalkylate fumarate [3,4]. The development of XSSs as biocatalysts would provide a direct route to access stereochemically complex, sp^3^-rich molecules from simple, achiral building blocks. Notably, when the native fumarate substrate is used as the olefin substrate, the resulting transformation produces 1,4-dicarbonyls—a privileged motif commonly found in drug scaffolds and valuable building blocks in organic synthesis [5]. Despite their importance, stereocontrolled synthesis of 1,4-dicarbonyls remains a significant challenge [6,7]. Exploring the substrate scope and stereoselectivity of XSS enzymes with non-native substrates, as well as engineering of these enzymes for improved function and expanded reactivity, could unlock powerful new biocatalytic strategies.

**Figure 1.**
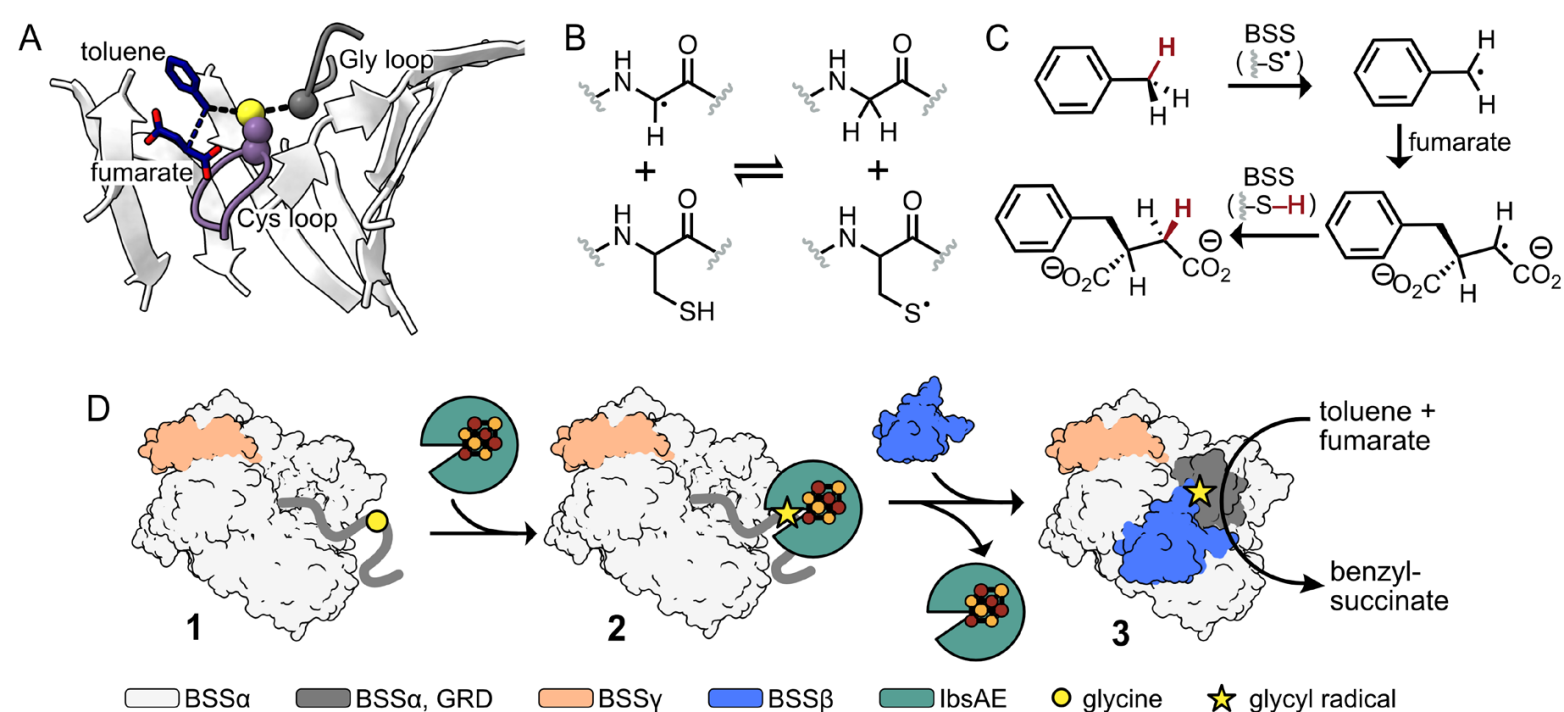
Benzylsuccinate synthase (BSS) catalyzes the addition of toluene to fumarate using amino acid–based radicals. (A) The Gly loop (dark grey) and the Cys loop (purple) of BSS are located in BSS’s 10-stranded β-barrel (white). Toluene (dark blue) is positioned between the catalytic Cys residue and a molecule of fumarate (dark blue). (PDB ID: 5BWE) [11] (B) After installation, the glycyl radical can form a transient thiyl radical on a Cys residue located within the Cys loop. (C) The thiyl radical abstracts an H atom from the methyl group of toluene. The resulting benzylic radical adds to fumarate, generating the succinyl radical, which re-abstracts the H atom from Cys to form *R*-benzylsuccinate and regenerates the thiyl radical for subsequent catalytic cycles. (D) Cartoon representation of BSS activation and hydroalkylation. **1:** BSSαγ adopts an open conformation, making the glycyl radical domain (GRD, dark grey) accessible for binding to IbsAE. **2:** When IbsAE (green) is added to reactions, it can bind to the GRD and subsequently install the glycyl radical (shown as a star), activating BSS for catalysis. **3:** Upon dilution of the activation reaction and addition of BSSβ, IbsAE is thought to dissociate. BSSβ stabilizes a closed conformation of BSSαγ, which is required for catalysis.

All members of the GRE superfamily feature a 10-stranded β/α-barrel that houses the enzyme’s active site, which contains both a Gly loop and a Cys loop (Figure 1A) [8]. A Gly radical, installed within the Gly loop by a radical *S*-adenosylmethionine (rSAM) activating enzyme (AE), initiates catalysis by forming a transient thiyl radical on a nearby Cys residue within the Cys loop (Figure 1A, B). This thiyl radical selectively abstracts a hydrogen atom from the benzylic position of toluene, generating a radical species that adds to fumarate via a Giese-type addition. The resulting succinyl radical re-abstracts a hydrogen atom from the catalytic Cys residue, regenerating the thiyl radical and yielding *R*-benzylsuccinate as the sole product (Figure 1C) [4]. A key advantage of XSS enzymes in biocatalysis is that once this Gly radical is installed, multiple rounds of turnover can occur with no additional cofactors or cosubstrates. While other GRE-AEs have been characterized, producing a soluble XSS-AE for in vitro radical installation using purified proteins remained a challenge until recently [1,8–11].

Here, we describe a method for the in vitro activation of XSS enzymes, specifically focusing on a well-characterized XSS, benzylsuccinate synthase (BSS) [12]. Unlike most GREs, which consist of a single subunit, BSS is composed of three distinct subunits—BSSα, BSSβ, and BSSγ (Figure 1D) [13,14]. We identified a soluble XSS-activating enzyme, 4-isopropylbenzylsuccinate synthase activating enzyme (IbsAE) [15], that can be expressed in *E. coli* as a soluble protein and demonstrates cross-reactivity with BSS [1]—an uncommon feature among characterized GREs [16]. Through optimization of activation conditions and hydroalkylation assays, we found that the BSSβ subunit inhibits BSS activation (i.e., radical installation) but is essential for hydroalkylation. Because of this finding, we optimized a protocol for producing BSSβ independent of BSSαγ [13], enabling its addition as a reagent in hydroalkylation reactions. We describe heterologous expression and purification protocols for BSSαγ, BSSβ, and IbsAE, which are carried out in an anaerobic chamber due to the oxygen-sensitive [4Fe–4S] clusters present in BSSγ, BSSβ, and IbsAE [13,14]. Recombinantly produced BSSαγ and IbsAE are used to install the Gly radical in BSSα (Figure 1D, **1–2**), with radical quantification performed via electron paramagnetic resonance (EPR) spectroscopy. Although SDS-PAGE can also be used to assess radical formation, we discuss the limitations of its use for accurate quantification. Once Gly radical is installed, the addition of BSSβ and substrates to activation reactions enables high assay yields (89%–97%) (Figure 1D, **2–3**). As XSSs that catalyze arylalkyl C–H bond activation generally share homologous αβγ subunits, we anticipate that our protocol can be adapted for other arylalkyl-SS enzymes as well. These protocols lay the groundwork for broader application of arylalkyl-SS enzymes in asymmetric biocatalysis. In addition, XSSs exist that activate alkane substrates [17]; however, they typically cluster separately from arylalkyl-SSs and contain additional accessory subunits [18]. We outline potential adaptations to extend our protocol for this distinct class of alkyl-XSSs.

## Materials and reagents

**Biological materials**

1. *E. coli* NiCo21(DE3) cells (New England BioLabs, catalog number: C2529H)

2. pET-28a-IbsAE. The gene for IbsAE (Uniprot ID A0A096ZNX5) was cloned into pET28a(+) (Novagen, kanamycin resistant) at restriction sites BamHI and HindIII. The N-terminal His-tag was removed, and a C-terminal His-tag was added. The full sequence can be found in Figure S1.

*Note: Both N-terminal and C-terminal His-tags produced soluble IbsAE; however, slightly higher yields were obtained when the tag was at the C-terminus.*

3. pET-DUET-BSSαγ. The gene for BSSα (Uniprot ID O68395) was cloned into the multiple cloning site 2 (MCS2), and BSSγ (Uniprot ID- O68394) was cloned into the multiple cloning site 1 (MCS1) of pET-DUET-1 (Novagen, ampicillin resistant). A C-terminal His-tag was added to BSSα. BSSγ remained untagged. The full sequence can be found in Figure S2.

4. pRSF-DUET-BSSβ. The gene for BSSβ (Uniprot ID O68396) was cloned into the MCS2 of pRSF-DUET-1 (Novagen, kanamycin resistant). An N-terminal His-tag was added to BSSβ. The full sequence can be found in Figure S3.

**Reagents**

**Reagents for protein expression and purification**

1. Lennox broth (LB) (RPI, catalog number: L24060)

2. Terrific broth (TB) (RPI, catalog number: T15000)

3. SOC outgrowth media (New England Biolabs, catalog number: B9020S)

4. Agarose LE (Gold Bio, CAS number: 9012-36-6)

5. Kanamycin sulfate (GoldBio, CAS number: 25389-94-0)

6. Ampicillin (GoldBio, CAS number: 69-52-3)

7. Iron (II) ammonium sulfate hexahydrate [Fe (NH_4_)_2_(SO_4_)_2_·6H_2_O] (Sigma-Aldrich, CAS number: 7783-85-9)

8. L-cysteine hydrochloride (Sigma-Aldrich, CAS number: 52-89-1)

9. Isopropyl β-D-1-thiogalactopyranoside (IPTG) (GoldBio, CAS number: 367-93-1)

10. HEPES [4-(2-Hydroxyethyl)piperazine-1-ethanesulfonic acid] (Sigma-Aldrich, CAS number: 7365-45-9)

11. Sodium chloride (NaCl) (Sigma-Aldrich, CAS number: 7647-14-5)

12. Imidazole (Sigma-Aldrich, CAS number: 288-32-4)

13. Glycerol (Fisher chemicals, CAS number: 56-81-5)

14. Lysozyme (Gold Bio, CAS number: 12650-88-3)

15. DNase I, bovine pancreas (Sigma-Aldrich, catalog number: 260913-25MU)

16. TALON^®^ metal affinity resin (Takara Bio, catalog number: 635502)

17. Ni Sepharose Excel (Cytiva, catalog number: 17371201)

18. Precision Plus Protein^TM^ All Blue standards, protein ladder (Bio-Rad, catalog number: 1610393)

19. 2-Mercaptoethanol (Sigma-Aldrich, CAS number: 60-24-2)

20. Bromophenol blue (Sigma-Aldrich, CAS number: 115-39-9)

21. Sodium dodecylsulfate (SDS) (RPI, CAS number: 151-21-3)

22. Tris(hydroxymethyl) aminomethane HCl (Tris-Cl) (RPI, CAS number: 1185-53-1)

23. 10× Tris/Glycine/SDS buffer (Bio-Rad, catalog number: 1610732)

24. Stain for SDS-PAGE (LC6065 SimplyBlue Safe Stain, catalog number: 465044)

**Reagents for radical installation and quantification**

1. 5-deazariboflavin (Santa Cruz Biotech., CAS number: 19342-73-5)

2. Dithiothreitol (DTT) (GoldBio, CAS number: 3483-12-3)

3. *S*-Adenosyl-L-methionine disulfate tosylate (AdoMet or SAM) (Ambeed, CAS number: 97540-22-2)

4. Potassium nitrosodisulfonate (Fremy’s salt) (Sigma-Aldrich, CAS number: 14293-70-0, catalog number: 220930)

**Reagents for hydroalkylation reactions and analysis**

1. Sodium fumarate dibasic (Sigma-Aldrich, CAS number: 17013-01-3)

2. Toluene (ACS reagent ≥99.5%) (Sigma-Aldrich, CAS number: 108-88-3)

3. Methanol (ACS reagent ≥99.8%) (Sigma-Aldrich, CAS number: 67-51-1)

4. 3-Chlorobenzoic acid (Sigma-Aldrich, CAS number: 535-80-8)

5. 2-Benzylsuccinic acid (Ambeed, CAS number: 884-33-3)

6. Dimethyl sulfoxide (DMSO) (Sigma-Aldrich, CAS number: 67-68-5)

7. LCMS-grade formic acid (Sigma-Aldrich, CAS number: 17013-01-3)

8. HPLC-grade acetonitrile (Sigma-Aldrich, CAS number: 108-88-3)

**Solutions**

1. LB media (see Recipes)

2. LB/agar media (see Recipes)

3. 50% glycerol stock (see Recipes)

4. TB media (see Recipes)

5. Kanamycin stock (see Recipes)

6. Ampicillin stock (see Recipes)

7. Iron (II) ammonium sulfate hexahydrate stock (see Recipes)

8. L-cysteine hydrochloride stock (see Recipes)

9. 1 M IPTG stock (see Recipes)

10. 1 M HEPES stock (see Recipes)

11. 5 M NaCl stock (see Recipes)

12. 2 M Imidazole stock (see Recipes)

13. Lysis/equilibration buffer (see Recipes)

14. Wash buffer (see Recipes)

15. Elution buffer (see Recipes)

16. Desalting/activation buffer (see Recipes)

17. 1× SDS-PAGE running buffer (see Recipes)

18. 10 mM 5-deazariboflavin stock (see Recipes)

19. 100 mM dithiothreitol (DTT) stock (see Recipes)

20. 36 mM AdoMet stock (see Recipes)

21. Fremy’s salt stock (see Recipes)

22. 100 mM fumarate stock (see Recipes)

23. 2 mM 3-chlorobenzoic acid stock (see Recipes)

24. 20 mM 2-benzylsuccinic acid stock (see Recipes)

25. SDS-PAGE loading dye (see Recipes)

**Recipes**

**1. LB media (1 L)**

**Table:** 

Reagent	Final concentration	Amount
LB	n/a	20 g
Milli-Q Water	n/a	To 1 L
Total		1 L

After mixing LB and water, autoclave on a standard liquid cycle (25 min sterilization time).

*Note: 1 L of media is typically made in a 2.8 L flask, capped with aluminum foil, and then autoclaved. Alternatively, media can be made in autoclavable bottles and autoclaved. In this case, empty flasks should be capped with aluminum foil and autoclaved as well.*

**2. LB/agar media (25 mL = 1 LB/agar plate)**

**Table:** 

Reagent	Final concentration	Amount
Agar	1.5% (w/v)	375 mg
LB media	n/a	To 25 mL
Antibiotics	Varies with antibiotic	25 μL

a. After mixing LB and agar, autoclave on standard liquid cycle (25 min sterilization time). Note that agar will not dissolve until autoclaved.

b. Gently swirl to thoroughly mix. Cool solution to ~50 °C and add appropriate antibiotic. Final concentration (ampicillin = 100 μg/mL; kanamycin = 50 μg/mL).

c. Gently mix the antibiotic into the solution and pour into the plate.

*Note: This recipe makes one agar plate; however, more can be made by scaling up the recipe.*

**3. 50% glycerol stock (1 L)**

**Table:** 

Reagent	Final concentration	Amount
Glycerol	50% (v/v)	50 mL
Milli-Q Water	n/a	50 mL
Total		100 mL

50% glycerol stock is used for making TB, buffers, and glycerol stocks of strains. The stock does not need to be autoclaved if used in TB (Recipe 4) or buffers (Recipes 13–15); however, if used for glycerol stocks of strains, autoclave on standard liquid cycle (25 min sterilization time).

**4. TB media (1 L)**

**Table:** 

Reagent	Final concentration	Amount
TB	n/a	47.6 g
50% glycerol stock	n/a	8 mL
Milli-Q Water	n/a	To 1 L
Total		1 L

After mixing TB, glycerol, and water, autoclave on a standard liquid cycle (25 min sterilization time).

*Note: 1 L of media is typically made in a 2.8 L flask, capped with aluminum foil, and then autoclaved. Alternatively, media can be made in autoclavable bottles and autoclaved. In this case, empty flasks should be capped with aluminum foil and autoclaved as well before use.*

**5. Kanamycin stock (10 mL)**

**Table:** 

Reagent	Final concentration	Amount
Kanamycin sulfate	1,000× (50 mg/mL)	500 mg
Milli-Q Water	n/a	To 10 mL
Total		10 mL

Aliquot as desired and store at -20 °C.

**6. Ampicillin stock (10 mL)**

**Table:** 

Reagent	Final concentration	Amount
Ampicillin	1,000× (100 mg/mL)	1 g
Milli-Q Water	n/a	To 10 mL
Total		10 mL

Aliquot as desired and store at -20 °C.

**7. Iron (II) ammonium sulfate hexahydrate stock (1 mL)**

**Table:** 

Reagent	Final concentration	Amount
Iron (II) ammonium sulfate hexahydrate	1,000× (75 mg/mL)	75 mg
Milli-Q Water	n/a	To 1 mL
Total		1 mL

a. Dissolve iron (II) ammonium sulfate hexahydrate in water (vortexing the solution can speed this up).

b. Pass stock through a 0.22 μm syringe filter to sterilize before use.

**8. L-cysteine hydrochloride stock (1 mL)**

**Table:** 

Reagent	Final concentration	Amount
L-cysteine hydrochloride	1,000× (23.5 mg/mL)	23.5 mg
Milli-Q Water	n/a	To 1 mL
Total		1 mL

a. Dissolve L-cysteine hydrochloride in water (vortexing the solution can speed this up).

b. Pass stock through a 0.22 μm syringe filter to sterilize before use.

**9. 1 M IPTG stock (10 mL)**

**Table:** 

Reagent	Final concentration	Amount
IPTG	1 M	2.383 g
Milli-Q Water	n/a	To 10 mL
Total		10 mL

Aliquot as desired and store at -20 °C.

**10. 1 M HEPES stock, pH 8.0 (1 L)**

**Table:** 

Reagent	Final concentration	Amount
HEPES	1 M	238.3 g
Milli-Q Water	n/a	To 1 L
Total		1 L

Adjust the pH to 8.0 by adding NaOH and store at 4 °C.

*Note: Dissolve HEPES in approximately 800 mL water, adjust the pH, then add water to reach a final volume of 1 L. The recipe can be scaled down as desired.*

**11. 5 M NaCl stock (1 L)**

**Table:** 

Reagent	Final concentration	Amount
NaCl	5 M	292.2 g
Milli-Q Water	n/a	To 1 L
Total		1 L

*Note: The recipe can be scaled down as desired.*

**12. 2 M imidazole stock, pH 8.0 (1 L)**

**Table:** 

Reagent	Final concentration	Amount
Imidazole	2 M	136.2 g
Milli-Q Water	n/a	To 1 L
Total		1 L

Adjust the pH to 8.0 by adding HCl and store at 4 °C.

*Note: Dissolve imidazole in approximately 800 mL of water, adjust the pH, then add water to reach a final volume of 1 L. The recipe can be scaled down as desired.*

**13. Lysis/equilibration buffer (500 mL)**

**Table:** 

Reagent	Final concentration	Amount
1 M HEPES stock	50 mM	25 mL
5 M NaCl stock	300 mM	30 mL
50% glycerol stock	5%	50 mL
Milli-Q Water	n/a	395 mL
Total		500 mL

a. Store in a cold room until use.

b. Sparge on a Schlenk line for 20 min with nitrogen gas before bringing into the anaerobic chamber.

*Note: This recipe is specifically for use with TALON resin. If using Ni Sepharose resin, imidazole should be added to a final concentration of 10 mM.*

**14. Wash buffer (500 mL)**

**Table:** 

Reagent	Final concentration	Amount
1 M HEPES stock	50 mM	25 mL
5 M NaCl stock	300 mM	30 mL
2 M imidazole stock	5 mM*	1.25 mL
50% glycerol stock	5%	50 mL
Milli-Q Water	n/a	393.75 mL
Total		500 mL

a. Store in a cold room until use.

b. Sparge on a Schlenk line for 20 min with nitrogen gas before bringing into the anaerobic chamber.

*Note: This recipe is specifically for use with TALON resin. If using Ni Sepharose resin, the final concentration of imidazole should be 20 mM.

**15. Elution buffer (500 mL)**

**Table:** 

Reagent	Final concentration	Amount
1 M HEPES stock	50 mM	25 mL
5 M NaCl stock	300 mM	30 mL
2 M imidazole stock	200 mM*	50 mL
50% glycerol stock	5%	50 mL
Milli-Q Water	n/a	345 mL
Total		500 mL

a. Store in a cold room until use.

b. Sparge on a Schlenk line for 20 min with nitrogen gas before bringing into the anaerobic chamber.

*Note: This recipe is specifically for use with TALON resin. If using Ni Sepharose resin, the final concentration of imidazole should be 300 mM.

**16. Desalting/activation buffer (500 mL)**

**Table:** 

Reagent	Final concentration	Amount
1 M HEPES stock	50 mM	25 mL
5 M NaCl stock	300 mM	30 mL
Milli-Q Water	n/a	445 mL
Total		500 mL

a. Store in a cold room until use.

b. Sparge on a Schlenk line for 20 min with nitrogen gas before bringing into the anaerobic chamber.

**17. 1× SDS-PAGE running buffer (1 L)**

**Table:** 

Reagent	Final concentration	Amount
10× Tris/Glycine/SDS buffer	1×	100 mL
Milli-Q Water	n/a	900 mL
Total		1 L

**18. 10 mM 5-deazariboflavin stock (2.66 mL)**

**Table:** 

Reagent	Final concentration	Amount
5-deazariboflavin	10 mM	10 mg
DMSO	n/a	To 2.66 mL
Total		2.66 mL

a. 5-deazariboflavin is light sensitive. Store in 100 μL aliquots in black Eppendorf tubes at -20 °C after freezing in liquid nitrogen. Alternatively, clear tubes can be wrapped in aluminum foil.

b. Transfer an aliquot to the anaerobic chamber in cold beads (-20 °C) prior to activation reactions.

**19. 100 mM dithiothreitol stock (DTT) (1 mL)**

**Table:** 

Reagent	Final concentration	Amount
Dithiothreitol	100 mM	15.4 mg
Desalting/activation buffer	n/a	To 1 mL
Total		1 mL

a. For desalting/activation buffer, see Recipe 16.

b. Store in 100 μL aliquots in Eppendorf tubes at -20 °C after freezing in liquid nitrogen.

c. Transfer an aliquot to the anaerobic chamber in cold beads (-20 °C) prior to activation reactions.

**20. 36 mM AdoMet stock (1 mL)**

**Table:** 

Reagent	Final concentration	Amount
AdoMet	36 mM	27.6 mg
Desalting/activation buffer	n/a	To 1 mL
Total		1 mL

a. For desalting/activation buffer, see Recipe 16.

b. Adjust pH with 10 M NaOH until the solution reaches neutral pH as indicated by pH strips.

c. Store in 100 μL aliquots in Eppendorf tubes at -20 °C after freezing in liquid nitrogen.

d. Transfer an aliquot to the anaerobic chamber in cold beads (-20 °C) prior to activation reactions.

**21. Fremy’s salt stock**

**Table:** 

Reagent	Final concentration	Amount
Fremy’s salt	X mM	~3–8 mg
Desalting/activation buffer	n/a	To 1 mL
Total		1 mL

a. For desalting/activation buffer, see Recipe 16.

b. Cycle a bottle of Fremy’s salt into the anaerobic chamber.

c. Add Fremy’s salt to an Eppendorf tube and add activation buffer. The solution should appear pale purple when near the desired final concentration range (~1–3 mM).

d. Transfer a small amount of this solution (20–50 μL) to a clean Eppendorf tube and bring this aliquot out of the anaerobic chamber to find the radical concentration by UV-Vis spectroscopy.

e. Measure the absorbance of the Fremy’s aliquot at 248 nm. The molar absorptivity is 1,690 M^-1^·cm^-1^. Use the Beer-Lambert law (*A*_248_ = *ε*_248_ *bC*, where *A*_248_ is absorbance at 248 nm, *ε*_248_ is molar absorptivity at 248 nm, *b* is the length of the light path, and *C* is the concentration of the solution) to find the final concentration of Fremy’s radical.

f. The anaerobic Fremy’s stock can then be used to make dilutions for EPR analysis.

*Notes:*

*1. Fremy’s stock is prepared fresh each time a standard curve is made for radical quantification.*

*2. While buffer conditions used to dissolve Fremy’s salt can vary, buffers with DTT or reductant are not compatible as they quench the radical.*

**22. 100 mM fumarate stock (1 mL)**

**Table:** 

Reagent	Final concentration	Amount
Sodium fumarate dibasic	100 mM	16.0 mg
Desalting/activation buffer	n/a	To 1 mL
Total		1 mL

a. For desalting/activation buffer, see Recipe 16.

b. Store in 100 μL aliquots in Eppendorf tubes at -20 °C after freezing in liquid nitrogen.

c. Transfer an aliquot to the anaerobic chamber in cold beads (-20 °C) prior to activation reactions.

**23. 2 mM 3-chlorobenzoic acid stock (10 mL)**

**Table:** 

Reagent	Final concentration	Amount
3-chlorobenzoic acid	2 mM	3.1 mg
Desalting/activation buffer	n/a	To 10 mL
Total		10 mL

a. For desalting/activation buffer, see Recipe 16.

b. Store at -20 °C.

**24. 20 mM 2-benzylsuccinic acid stock (10 mL)**

**Table:** 

Reagent	Final concentration	Amount
2-benzylsuccinic acid	20 mM	41.6 mg
Desalting/activation buffer	n/a	To 10 mL
Total		10 mL

a. For desalting/activation Buffer, see Recipe 16.

b. Store at -20 °C.

**25. SDS-PAGE loading dye (20 mL)**

**Table:** 

Reagent	Final concentration	Amount
2-Mercaptoethanol	5% (v/v)	500 μL
Bromophenol blue	0.02% (w/v)	4 mg
50% glycerol	30% (v/v)	12 mL
SDS	10% (w/v)	2 g
Tris-Cl	250 mM	0.788 g
Milli-Q Water	n/a	To 20 mL
Total	n/a	20 mL

**Laboratory supplies**

1. 1.7 mL Eppendorf tubes (e.g., Fisher Scientific, catalog number: NC1901967)

2. 15 mL conical tubes (e.g., Avantor, catalog number: 89039-666)

3. 50 mL conical tubes (e.g., Avantor, catalog number: 21008-242)

4. 14 mL sterile culture tubes (e.g., USA Scientific, catalog number: 5618-7262)

5. Standard Petri dish (e.g., USA Scientific, catalog number: 8609-1010)

6. 7.5% Mini-PROTEAN^®^ TGX^TM^ precast protein gel (e.g., Bio-Rad, catalog number: 4561023)

7. 4%–20% Mini-PROTEAN^®^ TGX^TM^ precast protein gel (e.g., Bio-Rad, catalog number: 4561093)

8. 10 mL Pierce^TM^ centrifuge columns (e.g., Thermo Scientific, catalog number: 89897)

9. PD-10 desalting column with Sephadex G-25 resin (e.g., Cytiva, catalog number: 17085101)

10. Amicon^®^ Ultra centrifugal filter, 10 kDa MWCO (e.g., Millipore Sigma, catalog number: UFC9010)

11. Amicon^®^ Ultra centrifugal filter, 50 kDa MWCO (e.g., Millipore Sigma, catalog number: UFC9050)

12. 96-well PCR plates (e.g., Alkali Scientific Inc., catalog number: PCFS96)

13. 1,000 μL micropipette tips (e.g., Tip One, catalog number: 1111-2721)

14. 200 μL micropipette tips (e.g., Tip One, catalog number: 1111-0706)

15. 10 μL micropipette tips (e.g., Tip One, catalog number: 1111-3700)

16. 0.22 μm syringe filter (e.g., Fisherbrand, catalog number: 09-720-004)

17. 2,800 mL Fernbach-style culture flask (e.g., Corning Life Sciences, catalog number: 4420-2XL)

18. Alkali Scientific Lab armor metal beads (e.g., Fisher Scientific, catalog number: 50-233-6206)

19. pH strips (e.g., Fisher Scientific, catalog number: M1095350001)

20. 96-well filter plates (Millipore, catalog number: MSGVN2250)

21. Seals for LCMS (BioChromato, catalog number: R80.120.00)

22. Parafilm (e.g., Parafilm M Laboratory Sealing Film, 4-inch × 125-foot roll, catalog number: 164996)

23. EPR tubes (e.g., 4 mm thin wall quartz EPR sample tube 250 mm L, Wilmad, catalog number: 707-SQ-250M)

24. Long-tip glass pipettes for transferring solutions to EPR tubes (e.g., Fisher Scientific, catalog number: 16-800-120)

## Equipment

1. Autoclave capable of sterilizing liquid media.

2. Expression equipment:

a. Static incubator for growing LB/agar plates at 37 °C (e.g., Thermo Scientific, catalog number: SHKE6000)

b. Horizonal shaking incubator for growing liquid cultures (e.g., Infors HT Multitron horizontal incubators with cooling, catalog number: MS022TS). Shaker should be able to rotate at 100–250 rpm. Cooling is necessary for expressions at 22 °C. Shaker decks should have clamps to hold 2.8 L Fernbach flasks or adhesive mats (e.g., “Sticky Stuff” from Infors HT, catalog number: MSTRAY)

3. Sonicator to break down the cell pellet inside the Coy (e.g., Fisherbrand^TM^ Model 705 Sonic Dismembrator, catalog number: FB705A110)

4. Thermo Sorvall X4 Pro centrifuge for cell pelleting and clarification (e.g., Thermo Scientific, catalog number: 75016052)

5. Small centrifuge to concentrate protein inside the anaerobic chamber (e.g., Eppendorf, model: 5430R, catalog number: 13690005)

6. UV/Vis spectrophotometer to determine concentrations of purified proteins and Fremy’s salt stocks (e.g., Thermo Multiskan Skyhigh microplate spectrophotometer, catalog number: 50-206-8594). Note that low volume measurements (2–10 μL) were performed using a μDrop plate (e.g., Thermo Scientific^TM^ μDrop, catalog number: 50-190-3760)

7. Anaerobic chamber. Two types (described below) were used throughout these studies, yielding consistent and reproducible results in each case:

a. Vinyl anaerobic chamber (e.g., Coy, Type B)

b. Glovebox workstation (e.g., MBraun, Unilab Pro)

8. Block heaters (e.g., VWR, model: Analog Heat Block, catalog number: 12621-104)

9. Freezer (-80 °C) to store protein (e.g., Thermo Scientific^TM^ TSX Series High-Performance Lab Refrigerators, catalog number: TSX50086APM)

10. Freezer (-20 °C) to store small molecule solutions (e.g., Fisher Scientific, model: Isotemp -20 Lab Freezer, catalog number: FBV20FPSA)

11. Water purification system (Thermo Scientific, model: Barnstead GenPure UV/UF-TOC with Bench Top x-CAD Plus, catalog number: 10-451-203)

12. Schlenk line for sparging buffers (ChemGlass, catalog number: AF-0452-01). Note that vacuum attachment is not necessary for these protocols. Nitrogen or argon gas can be used for sparging.

13. Protein electrophoresis equipment (Bio-Rad, catalog number: 1658004)

14. Waters Acquity Premier UPLC–MS system equipped with reversed-phase CORTECS Premier C18+ 1.6 μm 2.1 × 50 mm column

15. Mini-centrifuge (Onilab, model: D1008E)

16. EMX-Plus spectrometer for EPR (Bruker)

## Software and datasets

1. SkanIt UV/Vis software (Thermo Scientific)

2. Xenon EPR software (Bruker)

3. Empower LC-MS software (Waters)

## Procedure

**A. Expression and purification of BSSαγ, BSSβ, and IbsAE**

Expression and purification of BSSαγ and BSSβ have been previously described [1,11,13,14]. Expression and purification of IbsAE have been described in the original article [1].

Minor differences are noted below for the expression and purification of BSSαγ, BSSβ, and IbsAE.

1. Select the appropriate expression media based on the strain:

a. For expression of IbsAE, use TB media (see Recipe 4)

b. For expression of BSSβ or BSSαγ, use LB media (see Recipe 1)

2. Add the correct antibiotic to agar plates and liquid cultures according to the strain:

a. For IbsAE or BSSβ, supplement with 100 μL of kanamycin stock (see Recipe 5) to achieve a final concentration of 50 μg/mL.

b. For BSSαγ, supplement with 100 μL of ampicillin stock (see Recipe 6) to achieve a final concentration of 100 μg/mL.

3. Purifications can be performed using either TALON (Co^2+^) or Ni Sepharose (Ni^2+^) resins. While this protocol is designed for TALON, Ni Sepharose is a viable alternative. Key differences include higher (imidazole) in buffers (see Recipes 13–15) when using Ni Sepharose and higher loading capacity with Ni Sepharose. Purification on Ni Sepharose often results in slightly higher impurity levels.

**A1. Plasmid transformation**

1. Thaw 100 μL of chemically competent *E. coli* NiCo21(DE3) cells on ice for 5–10 min.

2. Mix cells gently with 10–50 ng of plasmid DNA (e.g., pET-DUET-BSSαγ, pRSF-DUET- BSSβ, pET-28a-IbsAE) using a pipette. Avoid rough mixing.

3. Incubate plasmid–cell mixture on ice for 30 min to facilitate plasmid adhesion to cell membranes.

4. Heat-shock cells at 42 °C for 45 s in a water bath to induce transient membrane permeability.

5. Immediately return to ice for 4 min to stabilize the cells.

6. Add 500 μL of SOC outgrowth media and recover at 37 °C for 1 h with shaking (220 rpm).

7. Plate 100 μL of the recovery culture onto LB agar plates supplemented with desired antibiotics.

8. Spread evenly using a sterile glass spreader, then incubate plates upside down at 37 °C for 16–18 h.

9. After 16–18 h, inspect plates for isolated, single colonies. An isolated, single colony will be picked for inoculation of the starter culture (see below).

*Note: Agar plates can be stored at 4 °C for up to 2 weeks.*

**A2. Starter culture**

1. Pour 100 mL of LB media into a 500 mL Erlenmeyer flask.

*Note: The starter culture will be used to inoculate expression cultures at a 1:10 starter-to-expression ratio, meaning 10 mL of starter culture should be used per 1 L of expression culture. The volume of starter culture can be adjusted accordingly.*

**Figure 2.**
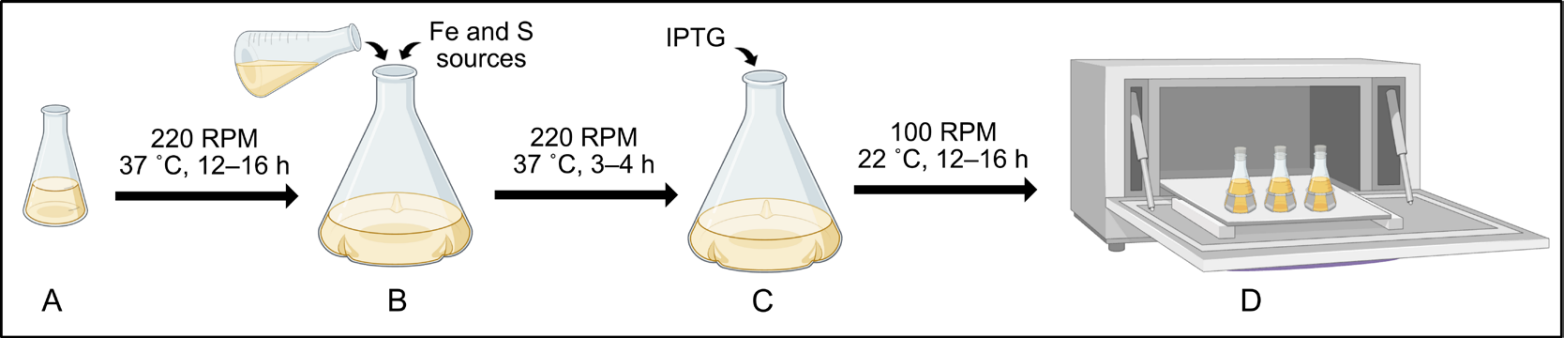
Protocol for bacterial growth and protein production. (A) Starter cultures are prepared by inoculation using a single colony from the transformation plate into LB media and are incubated overnight (12–16 h) to establish initial bacterial growth. (B) Expression cultures are inoculated with 10 mL of starter culture into fresh growth medium supplemented with iron (II) ammonium sulfate hexahydrate, L-cysteine, and the appropriate antibiotic, followed by incubation at 37 °C at 220 rpm. (C) Bacterial growth in expression cultures is monitored by measuring OD_600_. Once the OD_600_ reaches 0.6–0.8, expression cultures should be cooled at 4 °C for 30 min, after which IPTG is added to induce protein production. (D) Post-induction incubation of the expression culture requires reduced temperature (22 °C) and slower agitation (100 rpm) for proper protein folding and [4Fe–4S] incorporation.

2. Add 100 μL of the desired antibiotic to the media.

3. Inoculate the starter culture with a single colony from the transformation plate (step 9 from A1) using a sterile inoculation loop or pipette tip.

4. Incubate the starter culture overnight at 37 °C at 220 rpm for 12–16 h (Figure 2A).

**A3. Expression cultures**

1. In a 2.8 L Fernbach flask, add 1 mL of antibiotic stock solution to 1 L of pre-sterilized media (LB or TB).

2. Supplement the media with 1 mL of iron (II) ammonium sulfate hexahydrate stock and 1 mL of L-cysteine hydrochloride stock.

3. Inoculate the prepared sterile media with 10 mL of starter culture (Figure 2B).

4. Incubate cultures at 37 °C at 220 rpm until the OD_600_ = 0.6–0.8 (~2.5–4 h).

*Note: To measure OD_600_, use a sterile pipette tip to transfer 0.75–1 mL of bacterial culture to a clean cuvette. Measure the optical density at a 600 nm wavelength using a UV/Vis spectrophotometer. A higher OD_600_ corresponds to higher cell density.*

5. Transfer the cultures from 37 °C to 4 °C for 30 min.

*Note: Cultures can be placed in a cold room set to 4 °C or placed on an ice bath. Higher protein yields are obtained when this cooling step is incorporated into the expression procedure.*

**Figure 3.**
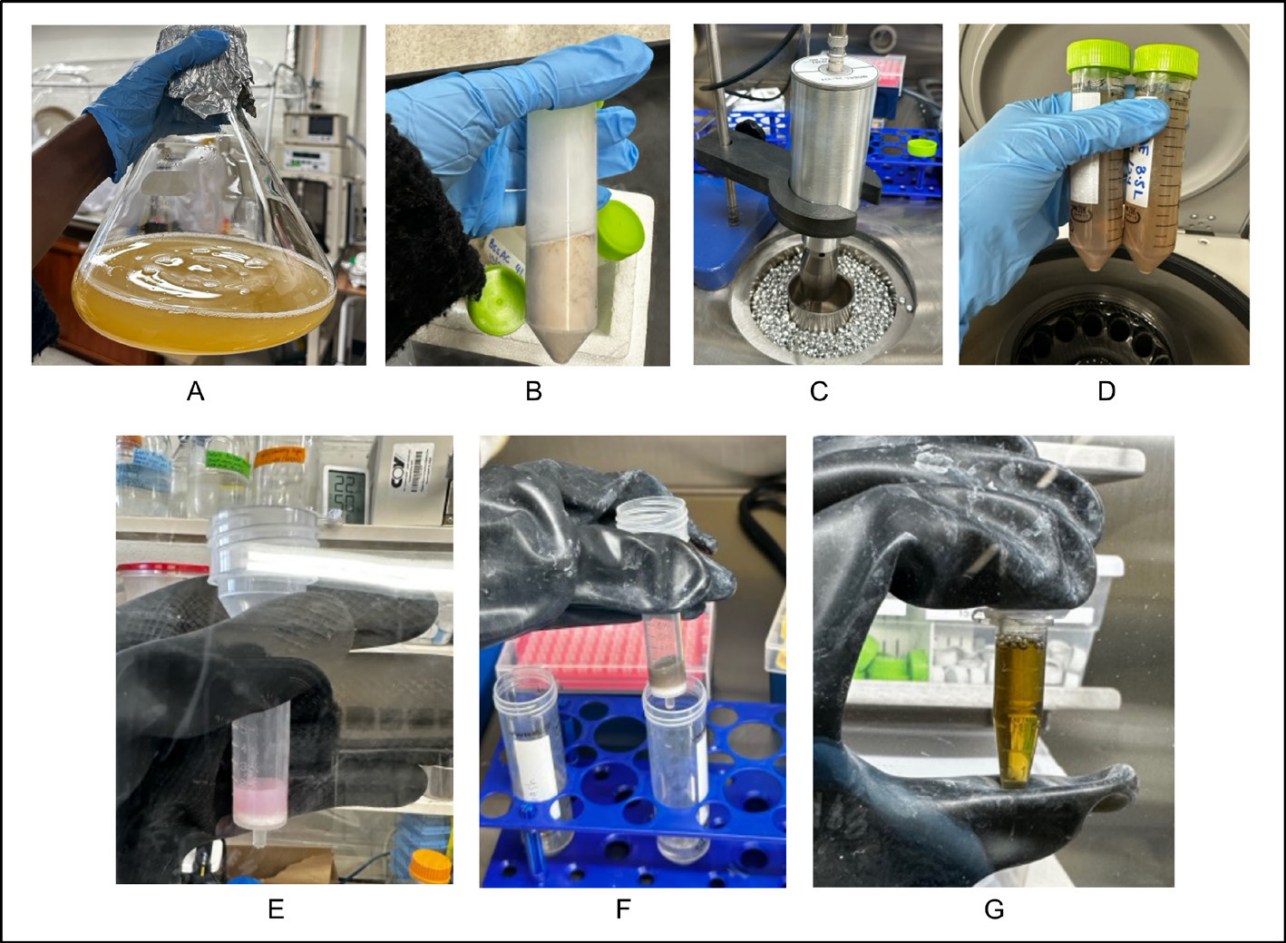
Protocol for anaerobic protein lysis and purification. (A) Expression culture prior to cell harvesting. (B) Harvested bacterial cell pellet after centrifugation, followed by immediate flash-freezing in liquid nitrogen and storage at -80 °C. (C) Cell lysis via sonication performed within an anaerobic chamber, with cell lysate embedded in cold beads to mitigate heat generation. (D) Clarified lysate after high-speed centrifugation (conducted outside the anaerobic chamber). (E) TALON resin prior to protein binding, displaying its characteristic pink color. (F) Protein-bound TALON resin exhibits a brownish color, indicating successful binding of the [4Fe–4S] cluster-containing protein to the resin. (G) Final purified protein sample after desalting and concentration, exhibiting the distinctive brown color typical of intact [4Fe–4S] clusters.

6. All four genes in this study (genes encoding BSSα, BSSγ, BSSβ, and IbsAE) are under the control of the T7 promoter. To induce gene expression, add 100 μL of IPTG stock for a final concentration of 100 μM (Figure 2C).

7. Reduce temperature to 22 °C and shaking speed to 100 rpm. Incubate for 12–16 h (Figures 2D and 3A).

*Note: Reduced shaking incorporates less oxygen into cell cultures, which is beneficial for [4Fe–4S] cluster incorporation into the proteins.*

8. Transfer expression cultures to centrifuge bottles.

9. Centrifuge at 2,700× *g* at 4 °C for 30 min to pellet cells.

10. Decant and discard supernatant.

11. Resuspend the cell pellet in 30 mL of lysis/equilibration buffer and transfer to a 50 mL conical tube.

*Note: We typically transfer cells from 2 L of expression culture into a single conical tube. The lysis and purification protocols were developed accordingly. If only 1 L of expression culture is used, the protocols for lysis and purification can be scaled down.*

12. Centrifuge again at 2,700× *g* at 4 °C for 20 min. Discard supernatant.

13. Flash-freeze pellets by submerging Falcon tubes in liquid nitrogen (Figure 3B).

14. Store at -80 °C until the purification step.

**A4. Anaerobic cell lysis**

**Critical:** Conduct lysis and purification in an MBraun anaerobic chamber (N_2_ atmosphere). Alternatively, use a Coy anaerobic chamber validated for equivalent performance (97.5% N_2_, 2.5% H_2_ atmosphere).

**Critical:** All the buffers used in lysis and purification are sparged with nitrogen (or argon) gas and subsequently cycled into the chamber. Keep buffers at 4 °C throughout lysis and purification. This can be done in an anaerobic chamber by keeping buffers in cold beads.

1. Cycle the cell pellets inside the anaerobic chamber while still frozen and let them thaw for 30–60 min.

*Note: Conical tubes must be cracked open during cycling of the antechamber.*

2. Weigh out approximately 40 mg of lysozyme into a serum bottle or equivalent container. Cover in parafilm. Puncture 2–3 small holes in the parafilm and cycle the bottle into the anaerobic chamber.

3. In the anaerobic chamber, add 40 mL of lysis/equilibration buffer to the lysozyme (final concentration of ~1 mg/mL) and gently mix. Add the resulting lysozyme solution to the cell pellet.

*Note: Adding more lysozyme is not problematic.*

4. Supplement with 25 μL of DNase I (2.5 MU/mL) and resuspend the cell pellet.

*Note: Using a spatula to mash the cell pellet can accelerate the resuspension step.*

**Optional:** The buffer can also be supplemented with an EDTA-free protease inhibitor cocktail to mitigate proteolytic degradation. Alternatively, phenyl methyl sulfonyl fluoride (PMSF) can be added as a serine protease inhibitor.

5. Cycle cold beads (at -20 °C) into the antechamber immediately before sonication.

6. Sonicate resuspended cells using the following parameters: 2 cycles of 2 min each, pulse: 2 s on, 2 s off, amplitude: 20.

*Notes:*

*1. Maintain samples in cold beads during sonication to prevent protein denaturation (Figure 3C).*

*2. Cold beads should be put at -20 °C the day prior to lysis to thoroughly cool them.*

*3. Cells can be sonicated directly in the conical tube or transferred to a metal beaker for sonication.*

7. Following cellular disruption by sonication, cycle the lysate out of the anaerobic chamber in a sealed tube for clarification (i.e., removal of insoluble cellular debris). In the centrifuge, spin at 14,000× *g* for 45 min at 4 °C.

*Note: Tubes for clarification should have an O-ring in the cap to seal when brought out of the anaerobic chamber. Oxygen will eventually permeate these tubes; therefore, there should be no delay between steps 7 and 8.*

8. Post-centrifugation, immediately return tubes to the anaerobic chamber.

9. Carefully aspirate the clarified supernatant, transferring it to clean Falcon tubes. Exercise caution to avoid the pelleted debris and lipid layer, as these will interfere with subsequent chromatographic steps. Clarified lysate should appear brown due to the [4Fe–4S] clusters, although this color will vary based on protein (Figure 3D).

*Note: If desired, lysate can be filtered through a 0.22 or 0.45 μm syringe filter.*

**A5. Anaerobic protein purification**

1. Pack 1 mL (settled volume) of TALON^®^ resin into a gravity-flow column (e.g., Pierce^TM^ centrifuge columns) (Figure 3E).

*Note: Ni Sepharose Excel resin can also be used. Imidazole concentrations in buffers will vary, and the binding capacity of resin will vary. We recommend using less volume of resin if using Ni Sepharose Excel resin.*

2. Equilibrate the resin with 15–20 column volumes (CV) of lysis/equilibration buffer. Allow the buffer to flow by gravity.

**Optional:** Buffers can also be passed through the resin by centrifugation. Low speeds (100× *g*) and short times (15–30 s) are required so that resin does not dry out.

3. Apply the clarified lysate (from step 9 of A4) to the equilibrated resin. Collect the flowthrough into clean 50 mL conical tubes for subsequent analysis by SDS-PAGE.

*Notes:*

*1. Monitor resin color. Unbound cobalt resin appears pink (Figure 3E); successful binding of Fe–S cluster-containing IbsAE, BSSαγ, and BSSβ induces a visible shift to dark brown upon passing all the clarified lysate through the resin (Figure 3F).*

*2. If the lysate has not been properly clarified, this step will be very slow by gravity.*

4. Wash the resin with 20 CV of wash buffer to remove weakly bound contaminants. Collect the wash after passing through the resin into clean 50 mL conical tubes for subsequent SDS-PAGE analysis.

5. Apply 500 μL of elution buffer. Discard this initial volume.

6. Immediately after the 500 μL of elution buffer has settled into the resin, place a clean conical under the column, and elute bound protein with 2.5 mL elution buffer. Collect the entire fraction, which will appear brown due to the [4Fe–4S] cluster.

**A6. Anaerobic buffer exchange via desalting chromatography**

1. Secure a PD-10 desalting column vertically in a Falcon tube. Wrap the column with parafilm to prevent premature buffer flow during cycling into the anaerobic chamber. Cycle into the anaerobic chamber.

2. Equilibrate the column with 25 CV (25 mL) of desalting/activation buffer. Allow the buffer to drain completely.

3. Slowly load the 2.5 mL immobilized metal affinity chromatography (IMAC) eluate onto the PD-10 resin bed. Allow the IMAC eluate to drain completely.

4. Place the desalting column into a clean conical tube for protein collection. Add 3.5 mL of desalting/activation buffer to the column. Collect the eluate as a single 3.5 mL fraction.

5. Transfer to a centrifugal filter. Centrifuge at 4,000× *g* in 5 min intervals at 4 °C until the retentate volume reaches the desired volume (typically between 250 and 500 μL).

*Notes:*

*1. Concentrate proteins using the appropriate centrifugal filters (50 kDa for BSSαγ and 10 kDa for IbsAE/BSSβ) inside the anaerobic chamber.*

*2. Convenient working concentrations range from 200 to 375 μM for IbsAE, from 150 to 300 μM for BSSαγ, and from 400 to 800 μM for BSSβ. Over-concentration can cause protein precipitation.*

6. Transfer concentrated protein into an Eppendorf tube and thoroughly mix. Save a small sample for SDS-PAGE analysis and for protein concentration determination. Dispense the remaining protein into 50–100 μL aliquots to minimize freeze-thaw cycles when using protein for reactions.

7. Cycle aliquots out of the anaerobic chamber and immediately immerse in liquid nitrogen.

8. Store at -80 °C.

**A7. SDS-PAGE to monitor protein purity**

**Figure 4.**
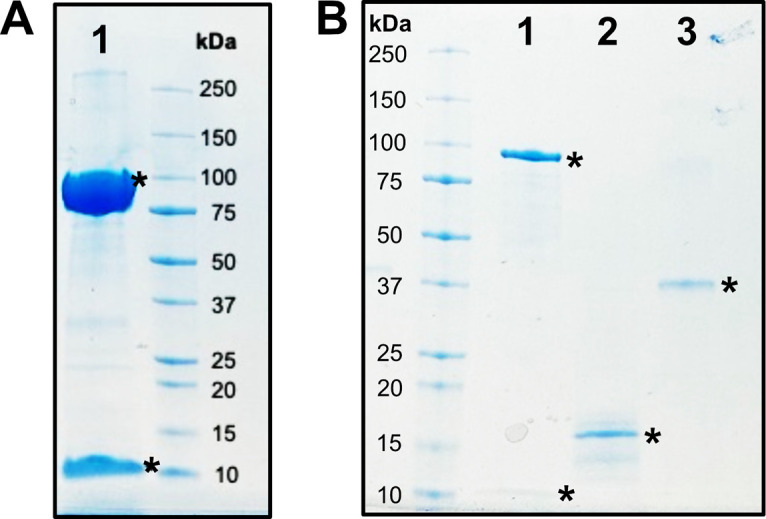
SDS-PAGE analysis of all protein components used in this study. (A) Lane 1: BSSαγ (99.0 and 6.9 kDa), concentrated. (B) Lane 1: BSSαγ (99.0 and 6.9 kDa), diluted. Lane 2: BSSβ (11.7 kDa). Lane 3: IbsAE (40.3 kDa). Stars denote proteins of interest.

1. Cycle fractions of flowthrough from IMAC (10 μL), wash from IMAC (10 μL), and final concentrated protein (1 μL) out of the box. Dilute concentrated protein with 9 μL of Milli-Q water.

2. Add 5 μL of SDS-PAGE loading dye to all fractions and mix gently.

3. Heat samples for 2 min at 95 °C prior to electrophoresis to denature protein.

4. Insert gel (4%–20%) into the electrophoresis gel holder and insert the gel holder into the tank.

5. Add 1× running buffer inside the electrophoresis gel holder to fully cover gel wells.

6. Load 2 μL of protein ladder and 6 μL of each sample in gel wells.

7. Connect the tank to the power supply and run gel at 270 V for 25 min.

8. Remove gel from plastic shell and place in a Tupperware container with dye. Microwave for 1 min and place on an orbital shaker for 10 min to stain the gel.

9. Destain the gel by replacing dye with Milli-Q water and shaking until gel is destained (~30 min).

10. Identify protein bands based on molecular weight (Figure 4) (99.0 and 6.9 kDa for purified BSSαγ, 40.3 kDa for purified IbsAE, and 11.7 kDa for purified BSSβ).

**A8. Protein quantification via UV spectrophotometry**

1. Cycle an aliquot of desalted, concentrated protein from step 6 of A6 out of the box for protein concentration determination via UV/Vis spectrophotometry.

2. Dispense 4 μL of purified protein sample into each of three wells. Load 4 μL of Milli-Q water into three separate wells as blanks.

3. Measure absorbance at 280 nm (A_280_) using a UV/Vis spectrophotometer.

4. Calculate the mean A_280_ for the sample and the blank.

5. Subtract the mean blank A_280_ from the mean sample A_280_.

6. Determine protein concentration using Beer-Lambert law:

*C* = (A_280_/*ε*_280_) * (1/*b*), where *C* = concentration (M), *ε*_280_ = extinction coefficient (M^-1^·cm^-1^) at wavelength 280 nm, *b* = path length (cm)

*Notes:*

*1. Extinction coefficients at wavelength 280 nm are as follows: IbsAE = 36,900 M^-1^·cm^-1^; BSSαγ = 141,150 M^-1^·cm^-1^; BSSβ = 17,990 M^-1^·cm^-1^.*

*2. Ensure the spectrophotometer is calibrated for microplate path length. Adjust the concentration calculation if using a pre-programmed microplate protocol.*

*3. Expected yields from purifications range from 6 to 8 mg/L for IbsAE, 6–10 mg/L for BSSαγ, and 5–7 mg/L for BSSβ.*

**B. Glycyl radical installation and quantification**

**B1. Enzyme activation (glycyl radical installation): 300 μL activation reaction**

1. Calculate the volumes of reagents to add for the activation reaction (Table 1). Note that the reaction volume for this example is 300 μL; however, activations have been successfully scaled from 25 μL to 2 mL. Required solutions include desalting/activation buffer (Recipe 16), DTT (Recipe 19), 5-deazariboflavin (Recipe 18), and AdoMet (Recipe 20). Required proteins can be made according to section A.

**Table 1.** Example of calculations for activation reaction (300 μL reaction)

Reagents	[Stock]	[Final]	Volume
Desalting/activation buffer	n/a	n/a	110.7 μL
DTT	100 mM	1 mM	3 μL
5-deazariboflavin	10 mM	50 μM	6 μL
IbsAE	197 μM	50 μM	76.2 μL
BSSαγ	164 μM	50 μM	91.6 μL
AdoMet	36 mM	1.5 mM	12.5 μL

2. Cycle all required materials into the anaerobic chamber in cold beads (-20 °C).

3. Add desalting/activation buffer to a 1.7 mL Eppendorf tube.

4. Add DTT (final concentration of 1 mM) and 5-deazariboflavin (final concentration of 50 μM). Mix gently.

5. Add freshly thawed IbsAE to the reaction mixture (final concentration of 50 μM) and mix gently with a pipette.

6. Add freshly thawed BSSαγ to the reaction mixture (final concentration of 50 μM) and mix gently with a pipette.

7. Initiate activation by adding AdoMet (final concentration of 1.5 mM).

8. Incubate the reaction in a metal heat block at 25 °C for 3–24 h.

*Notes:*

*1. Transfer IbsAE and BSSαγ into the anaerobic chamber right before the reaction.*

*2. Reducing the temperature significantly slows the rate of activation, while higher temperatures can cause the proteins to precipitate.*

*3. Reactions require exposure to light, and the fluorescent front-mounted lamp provided with the MBraun anaerobic chamber was found to be sufficient for this purpose, even when tubes were in the heat block. Too much light has been found to cause irreproducibility and lower levels of glycyl radical installation. When reactions are conducted within a Coy chamber, ambient light from the surrounding lab environment is sufficient.*

*4. Higher concentrations of 5-deazariboflavin (>200 μM) can also cause irreproducibility and lower levels of glycyl radical installation.*

*5. The activation reaction size can range from 25 μL to 2 mL.*

**Critical step:** Mix reactions gently by pipetting up and down three times without introducing any air.

**B2. SDS-PAGE to monitor activation**

**Figure 5.**
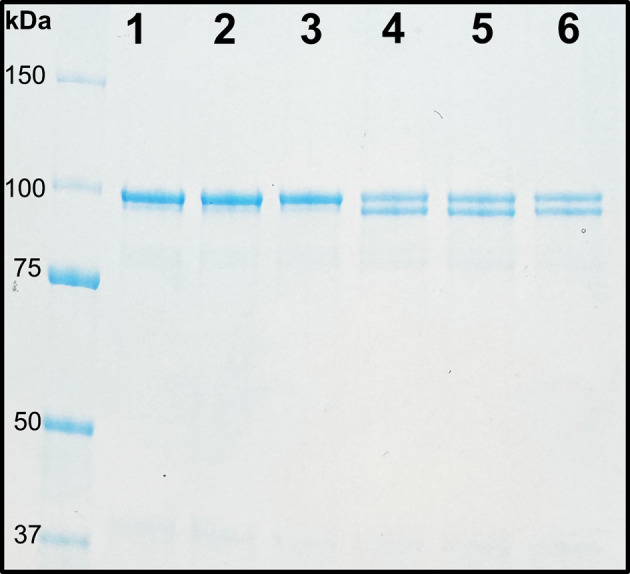
Exposure of glycyl radical to oxygen leads to protein cleavage. SDS-PAGE analysis of BSSα cleavage. Lanes 1–3: Unactivated BSSα as a control (99.0 kDa). Lanes 4–6: Activated BSSα exposed to oxygen, showing a double band corresponding to cleaved N-terminal fragment (93.3 kDa) and residual unactivated BSSα (99.0 kDa).

SDS-PAGE can be used as a method to analyze activation reactions. When the glycyl radical of BSSα is exposed to molecular oxygen, BSSα is cleaved into two polypeptide chains at the site of the radical (93.3 and 5.7 kDa). The amount of unactivated BSSα in a reaction (99.0 kDa) can be compared to activated BSSα (93.3 kDa) by SDS-PAGE (Figure 5) [19]. Although this method is useful for monitoring activation reactions and qualitatively assessing reaction progress, there are limitations to this method as a quantitative tool. Instead of directly measuring radical, here we are observing protein cleavage as a result of the radical. For the direct quantification of radical, see section B3.

1. Cycle 1 μL of activation reaction and 1 μL of unactivated BSSαγ out of the anaerobic chamber and expose to air.

*Note: For the unactivated BSSαγ control, it is recommended to use an activation reaction with a key reagent left out (e.g., AdoMet).*

2. Add 24 μL of Milli-Q water to both tubes containing activation reaction and unactivated BSSαγ.

3. Pipette 5 μL of each diluted sample from the previous step into new Eppendorf tubes.

4. Add 5 μL of SDS-PAGE loading dye to both tubes and mix gently.

5. Heat samples for 2 min at 95 °C prior to electrophoresis to denature protein.

6. Insert gel (7.5%) into the electrophoresis gel holder and insert the gel holder into the tank.

7. Add 1× running buffer inside of the electrophoresis gel holder to fully cover gel wells.

8. Load 2 μL of protein ladder and 4 μL of each sample into gel wells.

9. Connect the tank to a power supply and run gel at 270 V for 25 min.

10. Remove gel from plastic shell and place in a Tupperware container with dye. Microwave for 1 min and place on an orbital shaker for 10 min to stain the gel.

11. Destain the gel by replacing dye with Milli-Q water and shaking until gel is destained (~30 min).

12. A single band at 99.0 kDa for control should be observed for unactivated BSSαγ. A double band at 99.0 and 93.3 kDa should be observed for activated BSSαγ. The ratio of the 93.3 kDa band to the 99.0 kDa band provides an indication of the extent of activation.

**B3. EPR spectroscopy to quantitate glycyl radical**

**Figure 6.**
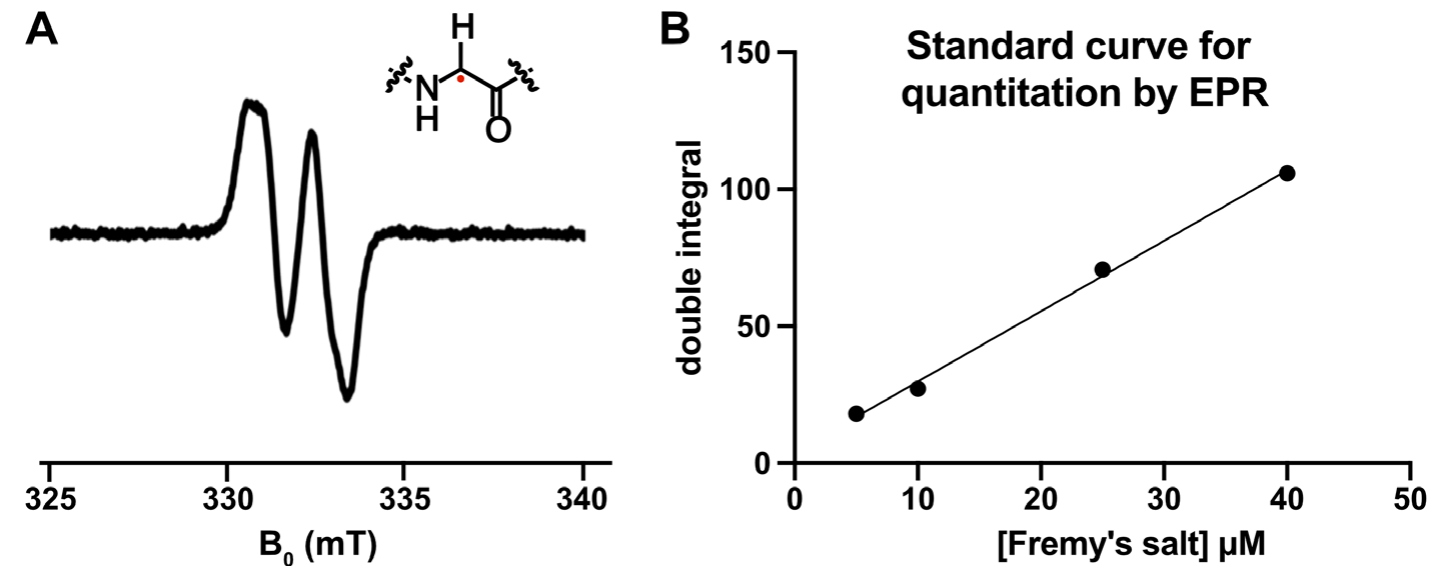
The glycyl radical in BSSα can be observed and quantitated using electron paramagnetic resonance (EPR) spectroscopy. (A) Representative EPR spectrum of activated BSSα, showing the characteristic doublet. (B) Known concentrations of Fremy’s radical can be plotted vs. the double integral of the resulting EPR signal to produce a standard curve for quantification. The curve can be used, along with the double integral of the unknown sample's signal, to determine the radical concentration of the unknown (e.g., glycyl radical).

As described previously for many other GREs [8,10,20], the glycyl radical can be directly observed using EPR spectroscopy (Figure 6A). Here, we describe how to determine the concentration of glycyl radical within BSSα using Fremy’s salt as a standard.

1. Set up activation reaction according to section B1 above. Typically, 250–300 μL reactions are recommended for the most accurate quantification.

*Note: Radical signal is dependent on the length and position of the sample in the resonator. By increasing reaction volume to 250–300 μL, the length of the sample will be longer than the resonator and remove the sample-to-sample discrepancies that could arise from shorter sample lengths.*

2. At the time of quantification, prepare a Fremy’s stock solution (Recipe 21) in the anaerobic chamber. Record the concentration of the stock. Use this stock to make a series of dilutions using desalting/activation buffer. The recommended range for these standards is 5–50 μM final concentration of Fremy’s salt.

3. Cycle in EPR tubes, glass transfer pipettes, pipette bulb, and liquid nitrogen into the anaerobic chamber.

4. Transfer 250–300 μL of activation reaction and Fremy’s standards into EPR tubes using a glass transfer pipette.

5. Slowly plunge the filled EPR tubes in liquid nitrogen to freeze.

*Note: Rapid freezing could break the tubes.*

6. Bring the samples out of the anaerobic chamber on liquid nitrogen.

7. Collect EPR spectra for activation and Fremy’s standards. Representative parameters: temperature = 120 K (note that temperatures from 77 to 120 K can be used for glycyl radical quantification with minimal change in signal), center field = 3320 G, sweep width = 200 G, sweep time = 60 s, receiver gain = 60 dB, modulation amplitude = 3 G, microwave power = 1.26 μW, time constant = 20.48 ms, conv. time = 89.96 ms.

*Notes:*

*1. It is highly recommended to optimize conditions. When optimizing conditions, collect spectra of samples at two microwave power settings that differ by a factor of four (e.g., 1.26 μW and 0.315 μW). The spectrum collected at lower power (e.g., 0.315 μW) should have a two-fold lower signal intensity. Use Xenon software to multiply the lower power spectrum by a factor of two. Overlay the two spectra. If the two spectra do not perfectly overlay, the conditions are not suitable for quantification.*

*2. Spectra for all samples must be collected using the exact same parameters; any variation compromises quantification.*

8. Use Xenon software to find the double integrals for spectra at optimized conditions for the sample and standards.

9. Plot the known concentration of Fremy’s salt against the double integral to create a standard curve (Figure 6B).

10. Use the standard curve and double integral of sample to calculate the concentration of glycyl radical.

**C. Using activated BSS for hydroalkylations**

**C1. Toluene addition to fumarate: 50 μL hydroalkylation reactions**

1. Calculate the volumes of reagents required for hydroalkylation (Table 2). Note that the reaction volume for this example is 50 μL; however, hydroalkylations have been successfully scaled from 25 to 100 μL. Required solutions include desalting/activation buffer (Recipe 16), fumarate stock (Recipe 22), and toluene. Required protein (BSSβ) can be made according to section A. Activation reaction is conducted according to section B.

**Table 2.** Example of calculations for hydroalkylation reaction (50 μL reaction)

Reagents	[Stock]	[final]	Volume
Desalting/activation buffer	n/a	n/a	5.7 μL
Fumarate	100 mM	2 mM	1 μL
Toluene	neat	1% (v/v)	0.5 μL
BSSβ	723 μM	40 μM	2.8 μL
Activated BSSαγ	50 μM	40 μM	40 μL

**Figure 7.**
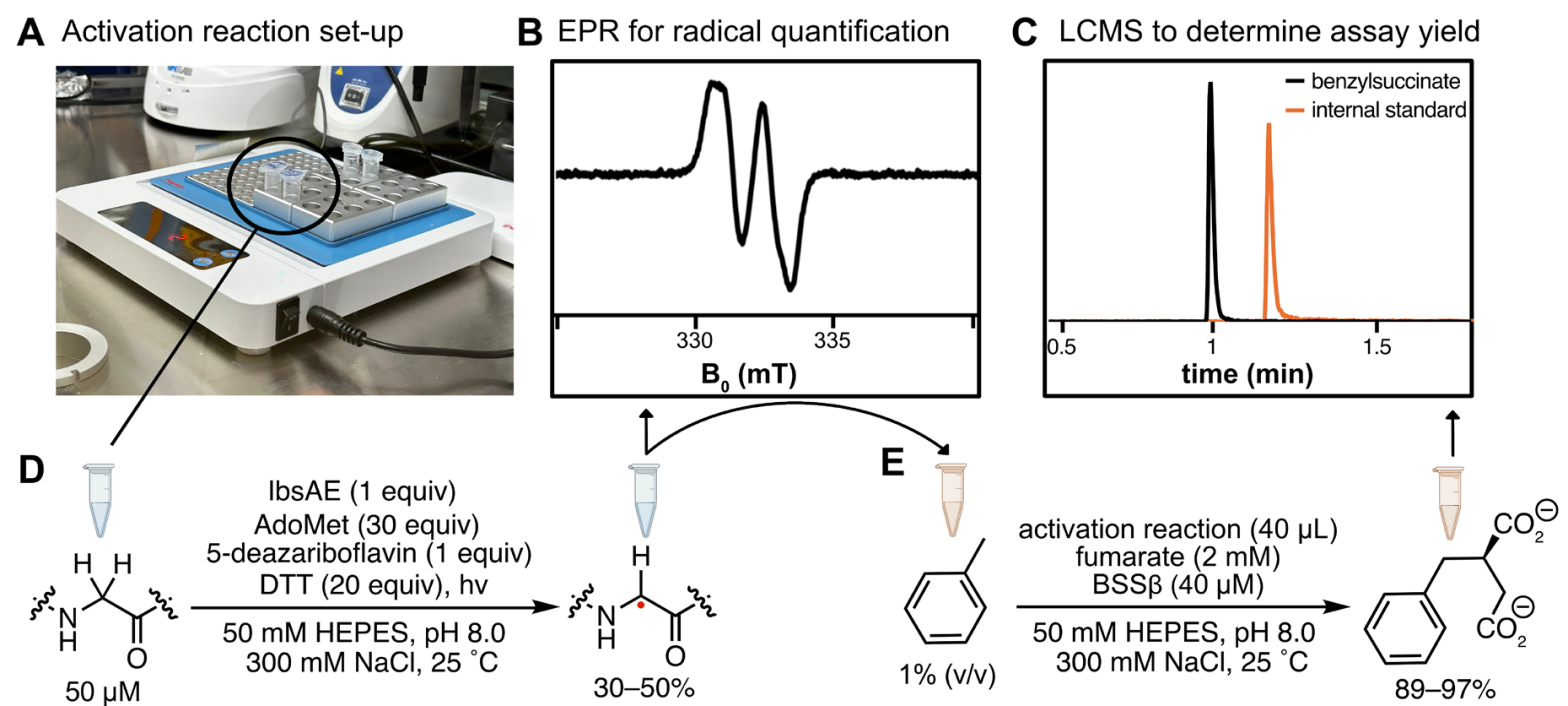
Overview of activation reactions and hydroalkylation reactions. (A) Activation reactions and hydroalkylation reactions are conducted in a heat block set to 25 °C within the anaerobic chamber. The transparency of the Eppendorf tubes allows sufficient light for activation reactions. (B) Activation reactions are quantified using EPR spectroscopy. (C) Hydroalkylation reactions are quantified using LC–MS. (D) Representative reaction scheme for activation reaction. (E) Representative reaction scheme for hydroalkylation reaction. Hydroalkylation reactions are initiated by adding activation reaction, containing the glycyl radical, n = 9.

2. Transfer all required reagents into the anaerobic chamber using pre-chilled (-20 °C) cold beads.

3. Begin by pipetting desalting/activation buffer into an Eppendorf tube.

4. Add fumarate and toluene. Mix gently.

5. Add BSSβ to the reaction mixture and mix gently.

6. Initiate the reaction by adding 40 μL of activation reaction to achieve a final concentration of 40 μM BSSαγ and mix gently.

7. Perform the reaction at room temperature and incubate on a heat block at 25 °C for approximately 1 h to achieve 89%–97% assay yield (Figure 7).

8. After approximately 1 h of incubation, remove the reaction from the anaerobic chamber and quench with 50 μL of methanol.

*Notes:*

*1. Transfer BSSβ into the anaerobic chamber right before the reaction.*

*2. All reagents must be anaerobic. Toluene can be stored in the anaerobic chamber in an airtight container, impenetrable to organic solvents. Alternatively, sparge toluene with an inert gas before each experiment and remove it from the chamber immediately afterward.*

*3. Assays are recommended to be performed in triplicate.*

**Critical step:** Mix reactions gently by pipetting up and down three times without introducing any air.

**C2. Sample preparation for LC–MS**

1. Add 2 mM 3-chlorobenzoic acid solution as an internal standard (I.S.) to the quenched reaction in a 1:1 (v/v) ratio relative to the reaction volume.

2. Centrifuge the reaction sample for 20 min at maximum speed in a centrifuge to pellet denatured protein.

3. Gently decant the supernatant using a pipette and dilute it 20-fold with Milli-Q water.

4. Filter the diluted solution through a 0.22 μm filter for LC–MS analysis. A syringe filter is sufficient if a small number of samples is being analyzed; however, a 96-well plate filter plate can be used for larger numbers of samples.

*Note: 96-well filter plates can be reused. We recommend passing Milli-Q water through them between each use.*

5. Calculate the enzymatic product concentration using the ratio of the area under the peaks corresponding to the [M-H] ion of benzylsuccinic acid (m/z = 207.1) and 3-chlorobenzoic acid (m/z = 155.1).

6. Determine the product concentration using a standard curve of 2-benzylsuccinic acid. Define the assay yield as [product] / [limiting reagent] × 100%.

**C3. Standard curve preparation for LC–MS**

1. Starting with 20 mM benzylsuccinic acid (Recipe 24), perform serial dilutions in Milli-Q water to generate the following concentrations: 2.5 mM, 1250 μM, 625 μM, 312.5 μM, 156.25 μM, 78.13 μM, 39.06 μM, 19.53 μM, 9.77 μM, and 4.88 μM.

2. Pipette 50 μL of each standard solution into separate wells of a 96-well plate.

3. To each well, add 3-chlorobenzoic acid stock (Recipe 23) in a 1:1 (v/v) ratio relative to the standard volume and mix gently.

4. Add 50 μL of methanol to each mixture (to match hydroalkylation reaction conditions) and mix gently.

5. Dilute the mixture 20-fold with Milli-Q water and filter through a 0.22 μm syringe filter or centrifuge using a 96-well plate filter.

6. To generate a standard curve, plot the ratio of the LC–MS peak area for the product ion to the peak area of the 3-chlorobenzoic acid ion (Figure 7C) against the known benzylsuccinic acid concentrations.

7. Use linear regression to generate the calibration curve.

**C4. LC–MS/MS method for assessing enzyme assays**

LC–MS data were obtained using a Waters Acquity Premier UPLC–MS system. For ionization, the mass spectrometer is equipped with an electrospray ionization (ESI) source. Separation of the samples was carried out on a reversed-phase CORTECS Premier C18+ 1.6 μm 2.1 mm × 50 mm column, using Milli-Q water with 0.1% formic acid as solvent A and acetonitrile with 0.1% formic acid as solvent B. The injection volume was 10 μL, flow rate was set to 0.8 mL/min, and gradient elution was performed as follows: t = 0 min, 5% B; t = 1.5 min, 95% B; t = 2.3 min, 95% B; t = 2.5 min 5% B; t = 3.0 min 5% B. MS detection was performed via ESI source in negative ionization mode. Product formation was monitored in selective ion recording (LC–MS-SIR) mode. Detection targeted two ions: benzylsuccinate ([M−H] = 207.1 m/z) and the internal standard 3-chlorobenzoic acid ([M−H] = 155.1 m/z). The ratios of peak areas were compared to the standard curve and used to quantify product yield.

## Validation of protocol

This protocol has been used and validated in the following research article:

• Andorfer et al. [1]. Development of an in vitro method for activation of X-succinate synthases for fumarate hydroalkylation. *iScience.*

For activations, n = 3. For hydroalkylations, n = 9.

These protocols were developed in Prof. Cathy Drennan’s lab at MIT [1]. All protocols herein were reproduced in Prof. Mary Andorfer’s lab at MSU (this work).

## General notes and troubleshooting

**General notes**

1. EPR spectroscopy should be used to quantify [glycyl radical] during activation experiments. While SDS-PAGE can indicate whether activation has occurred, it can overestimate [glycyl radical] levels. This discrepancy arises because EPR spectroscopy directly measures the radical itself, whereas SDS-PAGE assesses protein cleavage as an indirect indicator of radical. Protein cleavage is assumed to occur when the sample is intentionally exposed to oxygen upon removal from the anaerobic chamber; however, residual oxygen within the chamber can also cause premature cleavage, leading to inflated [glycyl radical] estimates.

2. Benzylsuccinate synthase (BSS) exhibits a remarkable ability to bind toluene, even in control experiments where no toluene is intentionally added. This occurs because BSS can scavenge toluene circulating in the anaerobic chamber. To minimize contamination, toluene should not be stored in the chamber unless sealed in an airtight container. Additionally, incorporating an activated carbon sorbent for solvent removal in the anaerobic chamber is recommended to further reduce toluene contamination (e.g., internal charcoal trap).

3. In activation reactions, light is required to excite 5-deazariboflavin; however, excessive light can lead to protein precipitation. Ambient light from the lab (for Coy chambers) or the box light (for MBraun chambers) is sufficient—additional lamps are unnecessary and, in some cases, detrimental. To maintain a constant temperature during activation, we use a metal heating block. Despite the block's enclosure, the transparency of Eppendorf tubes allows sufficient light to penetrate (Figure 7A).

4. This protocol is specifically designed for benzylsuccinate synthase (BSS) from *Thauera aromatica* [12]. In our original report, we demonstrated that both IbsAE and the protocol described here are also effective for the homologous isopropylbenzylsuccinate synthase (IBSS) [1,15]. Although BSS and IBSS act on different substrates, they cluster together in glycyl radical enzyme (GRE) sequence similarity networks alongside other known arylalkyl-succinate synthases (arylalkyl-SSs) that catalyze fumarate addition to benzylic carbons. In contrast, a separate class of XSSs—alkyl-succinate synthases (alkyl-SSs)—catalyzes fumarate addition to alkanes and clusters distinctly from arylalkyl-SSs [18]. We hypothesize that IbsAE and the protocol described here can be applied to other arylalkyl-SSs beyond BSS and IBSS. However, we anticipate that alkyl-SSs may require a different activating enzyme (AE) for glycyl radical installation, and the corresponding protocols may need to be adjusted. In particular, the order of addition of accessory subunits may vary. Though some modifications may be necessary, we expect this protocol will serve as a useful foundation for developing a method to activate alkyl-SSs as well.

5. Plastics should be placed in the anaerobic chamber well in advance of activation or hydroalkylation reactions, as they can retain residual oxygen. We recommend leaving plastics in the antechamber under static vacuum overnight before cycling them into the anaerobic chamber to minimize oxygen contamination.

6. All plasmids are available upon request.

**Troubleshooting**

Problem: Activation reactions appear cloudy, and/or glycyl radical is not being formed.

Possible cause: Small molecules or protein may be precipitating out of solution.

Potential solutions:

• Excessive light exposure: Too much light can cause protein precipitation; ensure only ambient light (e.g., Coy chamber room light or MBraun box light) is used, without additional lamps.

• Excessive heat: Overheating can cause protein precipitation. Use a controlled heating block to maintain a stable temperature.

• Incorrect reagent addition order: Follow a consistent and optimized order for adding reagents.

• Impure protein samples: Ensure thorough protein purification to prevent impurities from accelerating protein precipitation.

• Improper pH of small molecule stocks: Confirm that all solutions, including small molecule solutions (e.g., AdoMet), are properly pH-adjusted to neutral pHs.

Problem: The standard curve is not linear in MS analysis.

Possible cause: Detector saturation at higher concentrations may result in a plateaued or nonlinear response.

Potential solution: Dilute samples further with Milli-Q water to bring concentrations within the detector's linear range.

## Supplementary information

The following supporting information can be downloaded here:

1. Figure S1. pET-28a-IbsAE map and DNA sequence

2. Figure S2. pET-DUET-BSSαγ and DNA sequence

3. Figure S3. pRSF-DUET- BSSβ map and DNA sequence

The plasmid map figures were made with SnapGene^®^ software (from Dotmatics; available at snapgene.com)

## References

[1] Andorfer M. C., King-Roberts D. T., Imrich C. N., Brotheridge B. G. and Drennan C. L. (2023). Development of an in vitro method for activation of X-succinate synthases for fumarate hydroalkylation. iScience. 26(6): 106902 10.1016/j.isci .2023.106902 37283811 PMC10239695

[2] Chakraborty R. and Coates J. D. (2004). Anaerobic degradation of monoaromatic hydrocarbons. Appl Microbiol Biotechnol. 64(4): 437 446 446. 10.1007/s00253-003-1526-x 14735323

[3] Heider J., Szaleniec M., Martins B. M., Seyhan D., Buckel W. and Golding B. T. (2016). Structure and Function of Benzylsuccinate Synthase and Related Fumarate-Adding Glycyl Radical Enzymes. J Mol Microbiol Biotechnol. 26: 29 44 44. 10.1159/000441656 26959246

[4] Qiao C. and Marsh E. N. G. (2005). Mechanism of Benzylsuccinate Synthase: Stereochemistry of Toluene Addition to Fumarate and Maleate. J Am Chem Soc. 127(24): 8608 8609 8609. 10.1021/ja051972f 15954762

[5] Whittaker M., Floyd C. D., Brown P. and Gearing A. J. H. (1999). Design and Therapeutic Application of Matrix Metalloproteinase Inhibitors. Chem Rev. 99(9): 2735 2776 2776. 10.1021/cr9804543 11749499

[6] Lang K., Hu Y., Lee W. C. and Zhang X. P. (2022). Combined radical and ionic approach for the enantioselective synthesis of β-functionalized amines from alcohols. Nat Synth. 1(7): 548 557 557. 10.1038/s44160-022-00107-3 36713299 PMC9881596

[7] Friedmann T., Schuppe K., Laue M., Goldammer O. and Schneider C. (2024). Catalytic Enantioselective Synthesis of 1,4-(Hetero) Dicarbonyl Compounds through α-Carbonyl Umpolung. J Am Chem Soc. 147(2): 1948 1956 1956. 10.1021/jacs.4c14826 39812083 PMC11744765

[8] Backman L. R. F., Funk M. A., Dawson C. D. and Drennan C. L. (2017). New tricks for the glycyl radical enzyme family. Crit Rev Biochem Mol Biol. 52(6): 674 695 695. 10.1080/10409238.2017 .1373741 28901199 PMC5911432

[9] Külzer R., Pils T., Kappl R., Hüttermann J. and Knappe J. (1998). Reconstitution and Characterization of the Polynuclear Iron-Sulfur Cluster in Pyruvate Formate-lyase-activating Enzyme. J Biol Chem. 273(9): 4897 4903 4903. 10.1074/jbc.273 .9.4897 9478932

[10] Bodea S. and Balskus E. P. (2018). Purification and Characterization of the Choline Trimethylamine-Lyase(CutC)-Activating Protein CutD. In Methods in Enzymology. 606: 73 94 94. 10.1016/bs.mie .2018.04.012 30097105

[11] Funk M. A., Marsh E. N. G. and Drennan C. L. (2015). Substrate-bound Structures of Benzylsuccinate Synthase Reveal How Toluene Is Activated in Anaerobic Hydrocarbon Degradation. J Biol Chem. 290(37): 22398 22408 22408. 10.1074/jbc.m115 .670737 26224635 PMC4566215

[12] Coschigano P. (2002). Construction and characterization of insertion/deletion mutations of the tutF, tutD, and tutG genes of Thauera aromatica strain T1. FEMS Microbiol Lett. 217(1): 37 42 42. 10.1016/s0378-1097 (02)01051-0 12445643

[13] Li L., Patterson D. P., Fox C. C., Lin B., Coschigano P. W. and Marsh E. N. G. (2009). Subunit Structure of Benzylsuccinate Synthase. Biochemistry. 48(6): 1284 1292 1292. 10.1021/bi801766g 19159265 PMC2820103

[14] Funk M. A., Judd E. T., Marsh E. N. G., Elliott S. J. and Drennan C. L. (2014). Structures of benzylsuccinate synthase elucidate roles of accessory subunits in glycyl radical enzyme activation and activity. Proc Natl Acad Sci USA. 111(28): 10161 10166 10166. 10.1073/pnas.1405983111 24982148 PMC4104874

[15] Harms G., Rabus R. and Widdel F. (1999). Anaerobic oxidation of the aromatic plant hydrocarbon p-cymene by newly isolated denitrifying bacteria. Arch Microbiol. 172(5): 303 312 312. 10.1007/s002030050784 10550472

[16] Shisler K. A. and Broderick J. B. (2014). Glycyl radical activating enzymes: Structure, mechanism, and substrate interactions. Arch Biochem Biophys. 546: 64 71 71. 10.1016/j.abb .2014.01.020 24486374 PMC4083501

[17] Wilkes H. and Rabus R. (2020). Catabolic Pathways Involved in the Anaerobic Degradation of Saturated Hydrocarbons. In: Anaerobic Utilization of Hydrocarbons, Oils, and Lipids. 61–83. 10.1007/978-3-319-50391-2_4

[18] Levin B. J., Huang Y. Y., Peck S. C., Wei Y., Martínez-del Campo A., Marks J. A., Franzosa E. A., Huttenhower C. and Balskus E. P. (2017). A prominent glycyl radical enzyme in human gut microbiomes metabolizes *trans*-4-hydroxy-l-proline. Science. 355(6325): eaai8386. https://doi.org/10.1126/science.aai8386 PMC5705181 28183913

[19] Salii I., Szaleniec M., Zein A. A., Seyhan D., Sekuła A., Schühle K., Kaplieva-Dudek I., Linne U., Meckenstock R. U. and Heider J. (2021). Determinants for Substrate Recognition in the Glycyl Radical Enzyme Benzylsuccinate Synthase Revealed by Targeted Mutagenesis. ACS Catal. 11(6): 3361 3370 3370. 10.1021/acscatal.0c04954

[20] Leuthner B., Leutwein C., Schulz H., Hörth P., Haehnel W., Schiltz E., Schägger H. and Heider J. (1998). Biochemical and genetic characterization of benzylsuccinate synthase from *Thauera aromatica*: a new glycyl radical enzyme catalysing the first step in anaerobic toluene metabolism. Mol Microbiol. 28(3): 615 628 628. 10.1046/j.1365-2958 .1998.00826.x 9632263

